# Association between Three Mutations, F1565C, V1023G and S996P, in the Voltage-Sensitive Sodium Channel Gene and Knockdown Resistance in *Aedes aegypti* from Yogyakarta, Indonesia

**DOI:** 10.3390/insects6030658

**Published:** 2015-07-23

**Authors:** Juli Rochmijati Wuliandari, Siu Fai Lee, Vanessa Linley White, Warsito Tantowijoyo, Ary Anthony Hoffmann, Nancy Margaret Endersby-Harshman

**Affiliations:** 1 Pest & Environmental Adaptation Research Group, School of BioSciences, Bio21 Institute, 30 Flemington Rd, Parkville, The University of Melbourne, Victoria 3010, Australia; E-Mails: julir@student.unimelb.edu.au (J.R.W.); ronaldl@unimelb.edu.au (S.F.L.); vlwhite@unimelb.edu.au (V.L.W.); ary@unimelb.edu.au (A.A.H.); 2 Eliminate Dengue Project (EDP) Yogyakarta, Perumahan Sekip N-14, Bulaksumur, Yogyakarta 55262, Indonesia; E-Mail: wtantowijoyo@gmail.com

**Keywords:** pyrethroid, mosquito, bioassay, HRM, *Vssc*, *para*, *kdr*

## Abstract

Mutations in the voltage-sensitive sodium channel gene (*Vssc*) have been identified in *Aedes aegypti* and some have been associated with pyrethroid insecticide resistance. Whether these mutations cause resistance, alone or in combination with other alleles, remains unclear, but must be understood if mutations are to become markers for resistance monitoring. We describe High Resolution Melt (HRM) genotyping assays for assessing mutations found in *Ae. aegypti* in Indonesia (F1565C, V1023G, S996P) and use them to test for associations with pyrethroid resistance in mosquitoes from Yogyakarta, a city where insecticide use is widespread. Such knowledge is important because Yogyakarta is a target area for releases of *Wolbachia*-infected mosquitoes with virus-blocking traits for dengue suppression. We identify three alleles across Yogyakarta putatively linked to resistance in previous research. By comparing resistant and susceptible mosquitoes from bioassays, we show that the 1023G allele is associated with resistance to type I and type II pyrethroids. In contrast, F1565C homozygotes were rare and there was only a weak association between individuals heterozygous for the mutation and resistance to a type I pyrethroid. As the heterozygote is expected to be incompletely recessive, it is likely that this association was due to a different resistance mechanism being present. A resistance advantage conferred to V1023G homozygotes through addition of the S996P allele in the homozygous form was suggested for the Type II pyrethroid, deltamethrin. Screening of V1023G and S996P should assist resistance monitoring in *Ae*. *aegypti* from Yogyakarta, and these mutations should be maintained in *Wolbachia* strains destined for release in this city to ensure that these virus-blocking strains of mosquitoes are not disadvantaged, relative to resident populations.

## 1. Introduction

As in many tropical countries around the world, dengue continues to be a major public health concern in Indonesia. Data from the Indonesian Ministry of Health recorded that the number of dengue haemorrhagic fever cases in 2010 reached a peak of 156,086 with 1358 deaths [1]. Fewer cases have been reported since 2010, but numbers continue to be high (e.g., 101,218 cases with 736 deaths in 2013 [2]) and the disease burden on the population remains substantial. While dengue vaccine development has been an area of active research, the main way to prevent and control dengue virus transmission is to target its primary vector, the mosquito, *Aedes aegypti* (L.). Current dengue control in Indonesia focuses on community-based elimination of mosquito breeding sites [3,4,5,6], however chemical insecticides have been an important tool for more than 40 years of dengue vector control [6,7,8,9]. The two classes of insecticide that have been utilized intensively to prevent or reduce dengue transmission are organophosphates (temephos and malathion) and pyrethroids. Malathion fogging to control adult mosquitoes has been in use since 1973 [10] and, starting in 1980, was supplemented with use of temephos larvicides [7].

In the late 1980s, synthetic pyrethroids were introduced to control the dengue vector [11,12,13] and have been used widely since as a control measure to eliminate adult mosquitoes particularly during dengue outbreaks. Community-wide applications of pyrethroids have been used for ongoing seasonal vector control, and they have also been applied extensively at the household level. Specific pyrethroids advised by Indonesian vector borne diseases control programs include the adulticides cyfluthrin 50% EC; cypermethrin 25% ULV; lamdacyhalothrin 25% EC and permethrin and *S*-bioallethrin 10/1.5 OS; and alpha-*cypermethrin* [8].

Despite many ongoing efforts to combat dengue in Indonesia, control of the disease is facing many challenges. Perhaps the most important issue involves the development of insecticide resistance in *Ae. aegypti*, which probably has evolved due to a range of practices including frequent and/or indiscriminate application of insecticides. There are several ways that insect populations can become resistant to insecticides; resistance mechanisms include increased metabolic detoxification of the insecticide before it reaches its target site by a series of enzymatic reactions, as well as decreased sensitivity of the target site so that the insecticide does not bind and activate in the usual way [14,15].

The insecticide resistance status of *Ae*. *aegypti* in Indonesia has not been monitored on a regular basis, despite the strong dependence on insecticides for vector control. Pyrethroid resistance assays of three strains of *Ae*. *aegypti* from different areas in Indonesia demonstrated that a Bandung strain was resistant to permethrin and deltamethrin with RR_90_ (resistance ratios for 90% mortality) of 79.3 and 23.7, respectively [16]. Relatively weak resistance to permethrin (RR_90_ of 11.1) was detected in a strain from Palembang which was still susceptible to deltamethrin (RR_90_ of 2.2). In addition, a strain from Surabaya remained relatively susceptible to permethrin and deltamethrin with RR_90_ of 8.6 and 2.5, respectively [16]. Resistance to pyrethroids was studied in two laboratory reared strains (Namru and IPB) and a field collected strain (ITB) [17] and, in this case, all three strains exhibited some resistance to permethrin 92%, cypermethrin 92% and d-allethrin 93% indicated by high values of LT_90_. Strains resistant to permethrin from Larentuka and Semarang, Indonesia, were reported by Brengues *et al.* [18] and used in an investigation of pyrethroid resistance mechanisms.

Pyrethroids primarily affect both the peripheral and central nervous systems of insects by binding to a target site in the voltage-gated sodium channel or voltage-sensitive sodium channel (*Vssc*) within the nerve membrane [19]. They can be classified into two groups, Type I and Type II, based on their chemistry and effects. Type I pyrethroids typically exhibit low potency, knockdown and high repellency. They lack an α-cyano group at the phenoxybenzyl alcohol position and prolong opening of the sodium channel causing repetitive firing of the neuron [20]. Type II pyrethroids show a much higher potency with acute lethal effects. They contain an α-cyano-3-phenoxybenzyl alcohol moiety and prolong opening of the sodium channel causing membrane depolarization [20].

The intensive and sustained use of pyrethroids has led to the development of knockdown resistance (*kdr*) in many insect species [21]. *Kdr* is caused by a reduction in the sensitivity of sodium channels to pyrethroids, due to reduced binding of the insecticide at the target site [21,22]. The *kdr* phenotype is associated with a range of non-synonymous, single point mutations within the *Vssc* gene (*para*) which code for these channels in the transmembrane protein [23]. At least seven mutations have been detected in the *Vssc* gene of *Ae*. *aegypti*: V1023I (*i.e.*, Valine to Isoleucine amino acid change), V1023G, F1565C, I1018M, I1018V, S996P and D1794Y [24]. The most widely studied have been V1023I and F1565C. Du *et al.* [24] tested all seven *Vssc* mutations reported in *Ae. aegypti*, by developing a functional expression system for the sodium channel in *Xenopus* oocytes, but concluded that only three of these, V1023G, I1018M and F1565C are directly implicated in pyrethroid resistance.

V1023G has been shown to confer resistance to type I and II pyrethroids [25] and has been detected in *Ae. aegypti* from southeast Asia [26,27]. F1565C is thought to confer resistance to type I pyrethroids [25] and has been detected in *Ae*. *aegypti* from Thailand, Vietnam, the Caribbean [28,29] and recently in Brazil [30]. Mutation I1011M [24] only confers resistance to type I pyrethroids and is present in *Ae*. *aegypti* from South America and Martinique [18,31]. V1023I, the widely monitored mutation [29,32,33,34,35,36], did not alter channel sensitivity in the study by Du *et al.* [24].

In many insects, some *kdr* mutations are known to act in conjunction with others. An example is the first *kdr* mutation discovered in the house fly [26] which causes higher levels of resistance when another mutation is present (M918T super *kdr*) [19]. A recent study by Hirata *et al.* [37] suggested that S996P alone has no effect on the *Vssc* and pyrethroid resistance. However, it was shown to have a slight synergistic effect when it occurs in conjunction with V1023G and increases resistance to deltamethrin (Type II), but not to permethrin (Type I) [37]. V1023G alone reduced sensitivity of the *Vssc* to permethrin by 100-fold, whereas F1565C alone produced a 25-fold reduction. The same trend occurred for deltamethrin for V1023G, though the magnitude of the effect was smaller (2-fold). F1565C alone had no effect on the sensitivity of the *Vssc* to deltamethrin which agrees with the result of Du *et al.* [24]. Interestingly, the triple mutation (S996P, V1023G, F1565C), when created artificially and expressed in *Xenopus* oocytes for voltage clamp testing, appears to have synergistic effects, increasing the resistance effect of permethrin by 1100-fold and deltamethrin by 90-fold. V1023I did not alter channel sensitivity [24], but effects may be different if it occurs as a triple mutation as in the case above.

Brito *et al.* [34] crossed V1023I, F1565C homozygous resistant and homozygous susceptible lines of *Ae*. *aegypti* and found evidence that *kdr* is a recessive trait. Similar crosses made by Chang *et al.* [31] with V1023G, D1794Y homozygotes with susceptible lines suggested that the trait is incompletely recessive.

A number of techniques exist for detecting *kdr* mutations including allele-specific PCR [23,27,30,34,36,38]; Hot Ligation, a method that detects ligation between detector and reporter oligonucleotides when the detector is annealed to the SNP site using a thermal stable ligase and cycles of denaturing and hybridization to produce detectable qualities of ligated detector and reporter [29,35,39]; and Tetraplex, with two outer flanking primer to amplify a large fragment of target gene as a control and two inner allele specific primers in opposite directions to each other to generate smaller PCR products by forming PCR primer pairs with the outer primers [29,35]. All of these assays might be useful for routine diagnostic use. However, for large scale screening of samples, we need an assay that can detect these mutations of interest in a robust but faster manner than these conventional PCR assays.

**Figure 1:**
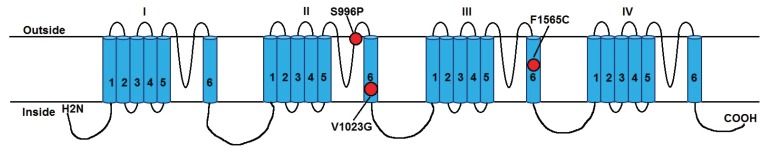
Positions of pyrethroid resistance-associated *Vssc* mutations of *Aedes aegypti* that are detected in this study. The schematic diagram shows the sodium channel protein indicating the four internally homologous domains (I–IV), each having six hydrophobic transmembrane helices (1–6). The mutations are numbered according to amino acid positions in the sodium channel gene of *Aedes aegypti* (diagram based on that of Du *et al.* [24]).

Research relating to point mutations in the target site of pyrethroid insecticides in *Ae*. *aegypti* is very limited in Indonesian material [18] and the *kdr* mutation status of *Ae*. *aegypti* is not known. Therefore we aimed to (1) develop high-resolution melt (HRM) genotyping assays to detect those mutations with the most evidence for causing pyrethroid resistance, F1565C, V1023G and S996P, in the voltage-sensitive sodium channel gene of *Ae*. *aegypti* (Figure 1) and use the assays to determine the frequency of these mutations in the mosquitoes around Yogyakarta; We also aimed to (2) investigate the association of the mutations both singly and in combination with resistance (as defined in bioassays); (3) determine the resistance status and *kdr* allelic composition of a *Wolbachia*-infected laboratory population with virus-blocking properties which has been outcrossed to field mosquitoes from Yogyakarta and which will be used to replace the natural population of *Ae*. *aegypti* as a dengue suppression technique; (4) provide information to assist with management of insecticide resistance; and (5) provide specific comparative resistance information to inform the release of *Wolbachia* mosquitoes in this city.

## 2. Experimental Section

### 2.1. Mosquito Samples

**Figure 2:**
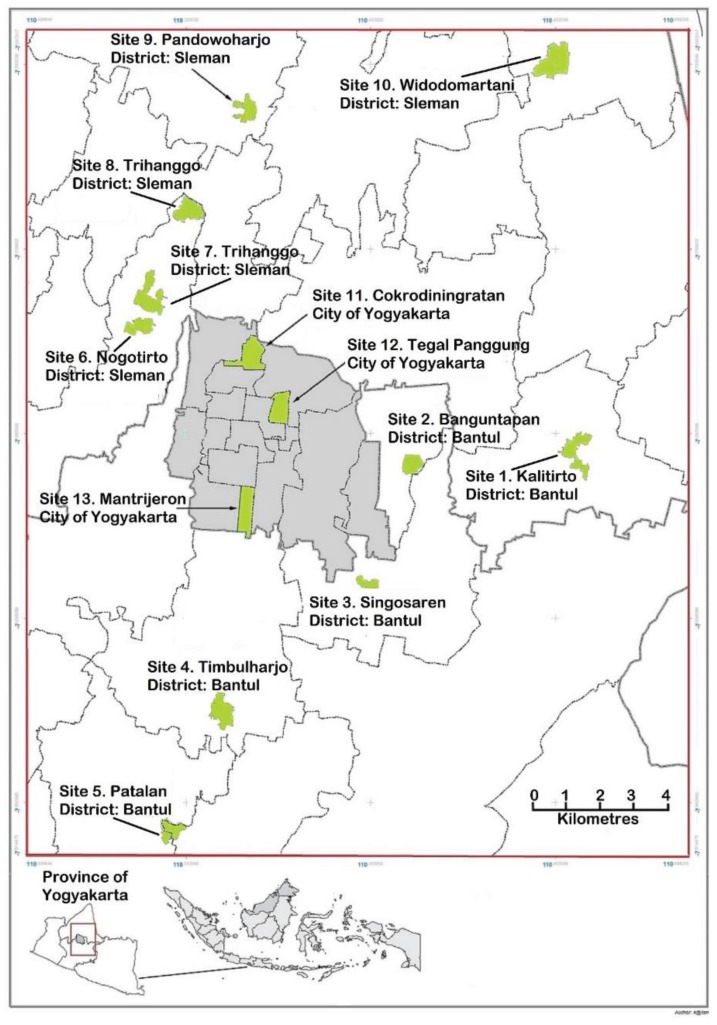
Map of Yogyakarta and its surrounding area showing the sites where samples of *Aedes aegypti* were collected.

Larvae of *Ae*. *aegypti* were collected using ovitrap buckets, 12.50 cm in diameter and 13.00 cm in height. About 900 mL fresh water was added to a level of 9 cm and a flannel oviposition strip (5 cm × 12 cm) was placed vertically with a part of it in the water. Fish food was added to attract female mosquitoes. Ovitraps were set up at ten “outer city” sites (Site 1 to Site 10) in Yogyakarta during November to December 2011 and these samples were described as Season 1. Season 2 comprised samples from five of the same “outer city” sites (Site 2, 3, 6, 7 and 8) collected in July 2012 and three “city” sites (Site 11, 12, and 13) collected in November 2012 (Figure 2). To ensure that there was a representation of mosquitoes from throughout each site, larvae were sourced from at least 100 indoor and 100 outdoor ovitraps placed at a site. Pupae were collected, separated into males and females, placed in cages and allowed to emerge as adults.

Three mosquito samples were used for bioassays of insecticides: Site 6, Site 8 and the *w*MelYog strain. For Site 6 and Site 8, samples were F_2_ from field-collected mosquitoes. The *w*MelYog strain is a laboratory strain made by crossing two uninfected field strains (derived from Site 6 and Site 8) with the *Wolbachia*-infected strain, *w*Mel [40,41] and undertaking repeated backcrossing for 6 generations to ensure that the infection was on the genetic background of the target strain.

### 2.2. Deltamethrin and Permethrin Bioassay

The standard WHO mosquito bioassay protocol was followed using bioassay tubes and insecticide-impregnated papers containing a diagnostic dose [42]. At least 150 non-blood fed, virgin females aged 3–5 days post emergence (four treatment replicates of 25 mosquitoes per tubes and two control replicates of 25 mosquitoes per tube) were evaluated for each insecticide from each site. The bioassay was repeated at a second time point.

Mosquitoes were exposed in a tube for 1 h to filter paper impregnated with deltamethrin (0.05%) or permethrin (0.75%) in a silicone oil solvent. The paper was obtained from Universiti Sains Malaysia (USM), Penang, Malaysia, a WHO collaborator. Control mosquitoes were exposed to papers impregnated with solvent (silicone oil) only. Knockdown was recorded after 1 h of exposure. All surviving mosquitoes were provided with 10% sugar solution, and final mortality was recorded at 24 h post-exposure.

### 2.3. DNA Extraction

Mosquito DNA was extracted using the Roche High Pure PCR Template Preparation kit (Mannheim, Germany; Cat. No. 11 796 828 001) in a final volume of 200 μL of elution buffer. A 10-fold dilution of the template DNA was prepared.

### 2.4. Tetra-Primer Assay to Genotype F1565C Mutation

The tetra-primer PCR procedure [29] was used to identify whether the F1565C mutation was present in the sodium channel gene of *Ae*. *aegypti* from Yogyakarta. PCR primer pairs designed by Harris *et al.* [29] were used to amplify exon 31 at domain III, subunit 6 of the voltage-sensitive sodium channel. They consist of a pair of flanking primers (AaEx31P and AaEx31Q) and two internal primers (AaEx31wt and AaEx31mut). The flanking primers were required to amplify a control band of 350 bp. Primer AaEx31wt was paired with AaEx31Q to genotype the “wild type” (phenylalanine allele) of 231 bp, whereas the combination of primer AaEx31mut and AaEx31P was used to genotype the mutant cysteine allele (AaEx31mut) of 163 bp. Forty-six samples were genotyped using this method.

Each PCR reaction was carried out in a total volume of 25 μL containing 2.5 μL 10× Reaction Buffer (MgCl_2_ free) (NEB); 1.25 μL 50 mM MgCl_2_; 4.00 μL 2.5 mM dNTPs (Bioline); 1.50 μL BSA (NEB) (10 mg/mL); 1.25 μL 10 μM Primer Forward; 1.25 μL 10 μM Primer Reverse; 0.50 μL Taq polymerase (NEB) (5 u/μL); and 5 μL of 1 in 10 diluted DNA template. Cycling conditions were as follows: initial denaturation of 95 °C for 5 min followed by 35 cycles of 94 °C for 30 s, 63 °C for 30 s, and 72 °C for 30 s, then a final elongation at 72 °C for 10 min. Each DNA template was assayed separately with all three primer combinations to yield either a 350 bp, 231 bp or 163 bp product. To visualize amplification, a 4 μL aliquot of each of the three PCR products were combined and mixed with 4 μL loading buffer and run on a 2% agarose gel where a single lane represents one individual typed for all three primer combinations. A 100-bp ladder (Hyperladder V, Bioline, Taunton, MA, USA) was used for sizing.

### 2.5. PCR Assay to Identify V1023G and S996P in Domain IIS6 of Vssc

Primers designed by Martins *et al.* [32] were used to identify mutations V1023G and S996P in the *Vssc* IIS6 region in *Ae*. *aegypti* from Yogyakarta. The primers were designed based on the alignment of the IIS6 region, partially covering exons 20 and 21, obtained from AaNav cDNA (GenBank accession No. AF534112) and the *Drosophila melanogaster* orthologous genomic DNA sequence (GenBank accession No. M32078): 5'-ACAATGTGGATCGCTTCCC-3' and 5'-TGGACAAAAGCAAGGCTAAG-3' [32].

Ninety mosquito samples from Yogyakarta with similar numbers each from Season 1 and Season 2 were amplified. The PCR was conducted in 40 μL [10× Reaction Buffer (MgCl_2_ free) (NEB) 4.0 μL; MgCl_2_ (50 mM) 1.2 μL; dNTPs (Bioline) (2.5 mM) 3.2 μL; 4.0 μL of each primer Forward and Reverse (10 μM); Taq polymerase (NEB) (5 u/μL) 1.0 μL, 4 μL DNA template (1 in 10 dilution) and 18.6 μL of ddH_2_0). Reactions were performed for 3 min at 94 °C for initial denaturation, followed by 35 cycles of 30 s at 94 °C for denaturation, 30 s at 60 °C for annealing, and 60 s at 72 °C for polymerase extension. To confirm amplification, aliquots of 10 μL of the PCR products were loaded onto 2.0% agarose gels.

### 2.6. DNA Sequencing

To confirm the results from the above PCR assays, 96 of the amplified samples (80 for V1023G and 16 for F1565C) were sequenced by Macrogen Inc. (Seoul, South Korea) in order to identify all mutations in *Ae. aegypti* in this region and to determine how the intron varies if multiple mutations from different exons are present. The results were used to ascertain if a set of primers could be designed to amplify the mutations in a real time PCR HRM assay. Sequencing data were analyzed using Geneious 7.0.5 Software (Biomatters Ltd., Auckland, New Zealand).

### 2.7. HRM Assays to Genotype F1565, V1023 and S996P Polymorphisms in the Para Gene

High-resolution melt (HRM) assays were developed to genotype the *F1565*, *V1023* and *S996P* polymorphisms in the *para* gene. The overall approach is as follows: Universal primers were designed flanking each target di-allelic polymorphism. Fluorescent (ResoLight™ Dye) PCR was performed in the LightCycler^®^ (Roche, Basel, Switzerland) 480 real time PCR machine. Genotypes of each target site were distinguished on the basis of their characteristic melt profiles.

**Figure A1:**
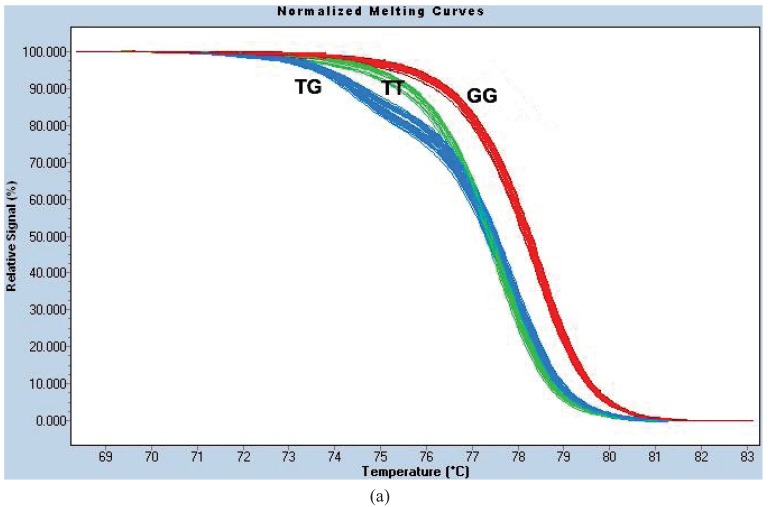

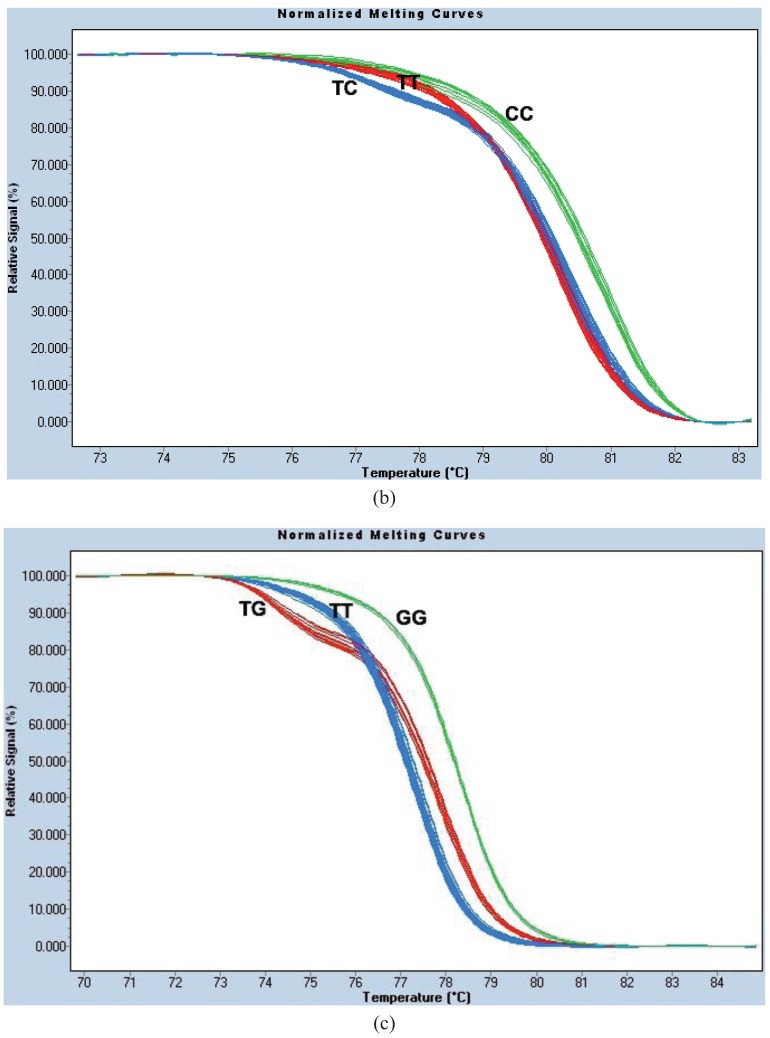
(**a**). HRM1023 assay of *Aedes aegypti*. The three genotypes (T/G, T/T and G/G) at site 1023 produce distinct melt curves; (**b**). HRM996 assay of *Aedes aegypti*. The three genotypes (T/C, T/T and C/C) at site 996 produce distinct melt curves; (**c**). HRM1565 assay of *Aedes aegypti*. The three genotypes (T/G, T/T and G/G) at site 1565 produce distinct melt curves. All genotypes were validated by amplicon sequencing.

The first target polymorphism, “site 1565”, is in the 24th codon of exon 31 in the *para* gene. Two non-synonymous polymorphisms are known: TTC (Phenylalanine) and TGC (Cysteine) (see Harris *et al.*, 2010 [29]). Genotyping primers, 5'-TACCTCTACTTTGTGTTCTTCATCATC-3' and 5'-GATTCAGCGTGAAGAACGACCCG-3', were placed adjacent to the target polymorphism. These primers produce an amplicon of 52 bp (Figure A1c). An additional primer pair, (5'-GTGGGAAAGCAGCCGATTCGCG-3' and 5'-CTAGGCCGTGGAATAGCTTTCAGC-3') was designed to yield a 245 bp product (encompassing target site 1565) for validation by Sanger sequencing. All primers for site 1565 are within exon 31.

The second target polymorphism, “site 1023”, is in the first codon of exon 21 of the *para* gene (VectorBase IDs: AAEL006109 and AAEL006109-PA). There are three known non-synonymous polymorphisms: GTA (Valine), GGA (Glycine) and ATA (Isoleucine) [23]. The ATA (Isoleucine) allele was not found in the 75 *para* sequences from 10 “outer city” sites (all sampled during November to December 2011 and five re-sampled in July 2012) and 3 “city” sites (all sampled in November 2012) from Yogyakarta mosquito populations. Our assay was therefore designed to differentiate between the GTA (Valine) and the GGA (Glycine) alleles. Genotyping primers (5'-GACAAATTGTTTCCCACCCGCACAG-3' and 5'-AAGCAAGGCTAAGAAAAGGTTAAG-3') flank the target site, producing an amplicon of 52 bp (Figure A1a). The priming site sequences were conserved among the 75 natural sequences from Yogyakarta.

The third target polymorphism, “site 996” is located in the P-region which links the membrane spanning segments S5 and S6 in Domain II of the *para* gene. The non-synonymous mutation occurs in the first codon position, changing serine (TCC) to proline (CCC) [43]. Genotyping primers used to amplify this site were 5'-CGGGTATTATGCGGCGAGTGGATC-3' and 5'-CCCACAAGCATACAATCCCACATGG-3' (Figure A1b). Amplicon size was 53 bp.

For site 1565, the 10 μL PCR reaction contained 2 μL of 1 in 10 diluted template DNA, 0.4 μL each of the primers at 10 μM, 1 μL of the ThermoPol reaction buffer (NEB Inc., Ipswich, MA, USA; Cat. No. B9004S), 0.064 μL of dNTP’s at 25 mM (Bioline, Alexandria, NSW, Australia; Cat. No. BIO-39029), 0.4 μL of MgCl_2_ (50 mM) (Bioline, Alexandria, NSW, Australia; Cat. No. BIO-21047), 0.25 μL of the LightCycler^®^ 480 High Resolution Melting Master (Roche, Mannheim, Germany; Cat. No. 04909631001), 0.01 μL of IMMOLASE^™^ DNA polymerase (10 u/μL) (Bioline, Alexandria, NSW, Australia; Cat. No. BIO-21047) and 5.476 μL of ddH_2_O (Honeywell, Burdick and Jackson; Muskegon, MI, USA; Cat. No. 365-4).

PCR amplification was carried out using the Roche LightCycler^®^ 480 system (384-well format). Thermo cycling steps were: 95 °C for 10 min, 20 cycles of 95 °C for 5 s, 65 °C (reduce 0.5 °C each cycle) for 15 s, 72 °C for 15 s, followed by an additional 20 cycles of 95 °C for 5 s, 55 °C for 15 s, and 72 °C for 15 s. Fluorescence information was captured at the end of each 72 °C step. PCR products were then subjected to HRM analysis. The HRM step involved heating the PCR products to 95 °C for 1 min, cooling to 40 °C for 20 s and then increasing the temperature to 65 °C. As the temperature increased from 65 to 95 °C, fluorescence data were recorded continuously. Melt curves were generated in the Gene Scanning module of the Roche LightCycler^®^ 480 software package. The parameter settings for melt curve normalization were: Pre-Melt Slider = 69.8–73.2 °C, Post-Melt Slider = 81.86–84.89 °C, Temperature Shift threshold = 0% and Sensitivity = 0.30 (Figure A1c). We termed this the “HRM1565 assay”.

For site 1023, the PCR reaction conditions were identical to those for 1565. The melt curves were normalized using the following settings: Pre-Melt Slider = 68.35–70.16 °C, Post-Melt Slider = 81.74–83.16 °C, Temperature Shift threshold = 0% and Sensitivity = 0.40. We termed this genotyping method the “HRM1023 assay” (Figure A1a).

For site 996, we used the same PCR protocol and thermocycling conditions as for HRM1023. The parameter settings for melt curve normalization were: Pre-Melt Slider = 73.69–76.18 °C, Post-Melt Slider = 83.53–84.86 °C, Temperature Shift threshold = 0% and Sensitivity = 0.40. This genotyping method is known as the “HRM996 assay” (Figure A1b).

Sanger sequencing of the HRM-genotyped individuals confirmed the accuracy of each of the assays. Assays were then used for screening of field samples and mosquitoes from bioassays with permethrin and deltamethrin.

### 2.8. Statistical Analysis

All data from the tetraprimer, HRM1565, HRM1023 and HRM996 screens of mosquitoes from Yogyakarta Season 1 and Season 2 were analyzed for site and season differences in allele frequencies using contingency tables with significance tested through the chi-square statistic. Permutation tests were used to determine significance where appropriate (*i.e.*, where expected values in cells were particularly low). For sites sampled in both seasons, a log linear analysis was performed to compare the variation in resistance allele frequencies between sites and seasons. All analyses were performed in IBM SPSS Statistics (IBM Corp., Armonk, NY, USA; 2013). The 95% binomial confidence intervals for allele frequencies were also computed.

To test for *kdr* genotype and insecticide resistance associations, mosquito resistance status from the bioassays was treated as columns and *kdr* genotypes from HRM assays were treated as rows. Genotypes of susceptible homozygotes and heterozygotes were collapsed into one group as the heterozygote is also expected to have a phenotype close to susceptible because of the incomplete recessive effect of *kdr* mutants on resistance (as opposed to these mutations being incompletely dominant). Odds ratios (with 95% confidence intervals) [44] were calculated to indicate the odds of an individual being resistant if it carried a copy of the putative resistance mutation. The ratio is generated from a 2 × 2 table (two genotypes *vs.* two effects) [OR = (axd)/(bxc)] [45]. An odds ratio of 1 indicates that there is no relationship between resistance and the genotype under investigation. If 95% confidence intervals of the odds ratio do not span the value “1” then this suggests that the genotype is associated with resistance. We also analyzed significance by testing the association between the putative resistance genotypes and the resistance phenotype using Fisher’s exact tests [45]. Odds ratios were also used to determine whether mutations in combination in an individual were more likely to be associated with resistance than mutations occurring singly.

## 3. Results

### 3.1. Assay Development for F1565C

A tetra-primer PCR assay [29] was used initially to determine the presence of the F1565C mutation in Yogyakarta Season 1 and Season 2 mosquito samples. The PCR assay showed strong, clean bands of the expected product sizes of 350 bp and 231 bp for susceptible homozygous (FF) individuals, and an additional band of 163 bp for the susceptible heterozygote. We did not find individuals with only 350 bp and 163 bp bands (resistant homozygote, CC) (data not shown). Sixteen samples from the PCR assay were sequenced to confirm the genotypes. Eleven were confirmed as susceptible homozygotes (FF) and four were confirmed as heterozygotes (FC). However, one individual scored as a heterozygote in the PCR assay was shown to be a resistant homozygote (CC) when sequenced. This discrepancy in genotyping prompted development of the HRM assays described in Section 2.7.

### 3.2. DNA Sequencing of V1023G and S996P

DNA sequencing confirmed that 62 out of 75 individuals from Yogyakarta Season 1 and Season 2 were homozygous for V1023G due to a T/G nucleotide mutation. This meant that a very high percentage of individuals were homozygous for the putatively resistant G allele, consisting of 70.27% of individuals from Season 1 and 94.74% from Season 2 (Table 1). Eleven individuals were heterozygous and only two were homozygous for the putatively susceptible V1023 allele (Table 1). Both resistant homozygotes and susceptible heterozygotes showed very limited sequence variation (less than 1% both within and between groups). Only two susceptible homozygotes were detected and these showed approximately 2% sequence variation. Resistant homozygotes plus susceptible heterozygotes showed very high sequence variation (approximately 12%) (including detected indels) with susceptible homozygotes for site 1023 (data not shown).

Sequencing results also showed evidence of the S996P mutation thought to be associated with resistance. However, the percentage of individuals homozygous for the putative resistant C allele was lower than for V1023G, and consisted of 13.51% of the population in Season 1, and 21.05% in Season 2.

**Table 1:** Sequencing results showing resistance allele frequencies of *Aedes aegypti* samples from ten sites in Yogyakarta Season 1 and eight sites in Yogyakarta Season 2 for the *Vssc* V1023G and S996P mutations (genotypes in bold have been associated with insecticide resistance).

Season	N	V1023 Sequence			S996P Sequence		
		T/T	G/T	G/G	T/T	T/C	C/C
1	37	2	9	26	17	15	5
2	38	0	2	36	11	19	8

### 3.3. Assay Development for V1023G and S996P

The sequencing results for both assays above were used to design primers that could genotype the mutations F1565C, V1023G, and S996P by HRM analysis.

#### Screening of Field Samples

The frequencies of F1565C, V1023G, and S996P polymorphisms across Yogyakarta estimated with assays HRM1565, HRM1023, HRM996 are shown in Table 2 for Yogyakarta Season 1 samples and Table 3 for Yogyakarta Season 2 samples.

The homozygous resistant genotype 1565C/1565C was rare, only one was detected in a total of 151 samples; the homozygous 1023G/1023G individuals predominated at 58% in Season 1 (Table 2). The frequency of the 996P/996P homozygote was 9.27%. Based on contingency analysis, there were no significant differences in frequencies of the putative resistance allele F1565C (χ^2^ = 15.895, df = 9, *p* = 0.06) and frequencies of V1023G (χ^2^ = 14.938, df = 9, *p* = 0.93) between sites in Season 1. However, a significantly different frequency of resistance was found among sites for the S996P allele (χ^2^ = 30.709, df = 9, *p* < 0.001).

**Table 2:** HRM assay results for *Vssc* F1565C, V1023G and S996P mutations of *Aedes aegypti* from Yogyakarta Season 1.

Site	Genotype F1565C			Freq. of C Allele (95% CI)	Genotype V1023G			Freq. of G Allele (95% CI)	Genotype S996P			Freq. of P allele (95%)
	FF	FC	CC		VV	VG	GG		SS	SP	PP	
1	15	0	0	0 (0, 0.116)	0	10	5	0.667 (0.496, 0.827)	13	2	0	0.067 (0.221, 0.008)
2	12	2	1	0.133 (0.039, 0.307)	1	3	11	0.833 (0.693, 0.944)	5	6	4	0.467 (0.286, 0.657)
3	14	1	0	0.033 (0.001, 0.172)	2	5	8	0.7 (0.532, 0.853)	8	2	5	0.4 (0.236, 0.584)
4	15	0	0	0 (0, 0.116)	3	8	4	0.533 (0.359, 0.716)	10	5	0	0.167 (0.058, 0.347)
5	14	1	0	0.033 (0.001, 0.172)	0	5	10	0.833 (0.693, 0.944)	10	4	1	0.2 (0.080, 0.386)
6	21	0	0	0 (0, 0.116)	1	9	11	0.738 (0.600, 0.861)	13	7	1	0.214 (0.106, 0.368)
7	15	0	0	0 (0, 0.116)	1	3	11	0.833 (0.693, 0.944)	5	10	0	0.337 (0.180, 0.528)
8	12	3	0	0.1 (0.022, 0.265)	2	4	9	0.733 (0.570, 0.877)	8	6	1	0.267 (0.128, 0.459)
9	9	1	0	0.05 (0.001, 0.249)	1	2	7	0.8 (0.617, 0.943)	2	6	2	0.5 (0.291, 0.728)
10	13	2	0	0.068(0.221, 0.008)	1	2	12	0.867 (0.738, 0.962)	13	2	0	0.067 (0.008, 0.221)
**Total**	**140**	**10**	**1**	**0.040** (**0.021**, **0.068**)	**12**	**51**	**88**	**0.752** (**0.702**, **0.799**)	**87**	**50**	**14**	**0.258** (**0.211**, **0.311**)

* Some individuals were not genotyped successfully for all three mutations.

**Table 3:** HRM assay results for *Vssc* F1565C, V1023G and S996P mutations of *Aedes aegypti* from Yogyakarta Season 2.

Site	Genotype F1565C			Freq. of C Allele (95% CI)	Genotype V1023G			Freq. of G Allele (95% CI)	Genotype S996P			Freq. of P Allele (95% CI)
	FF	FC	CC		VV	VG	GG		SS	SP	PP	
2	31	9	0	0.113 (0.053, 0.203)	0	9	31	0.888 (0.814, 0.947)	21	15	4	0.313 (0.216, 0.426)
3	36	3	1	0.063 (0.021, 0.140)	1	9	30	0.863 (0.782, 0.929)	16	18	6	0.375 (0.273, 0.490)
6	36	3	1	0.063 (0.021, 0.140)	1	14	25	0.800 (0.708, 0.881)	32	7	1	0.313 (0.216, 0.426)
7	35	5	0	0.063(0.021, 0.140)	0	11	29	0.863 (0.782, 0.929)	16	18	6	0.375 (0.273, 0.490)
8	37	2	0	0.026(0.003, 0.090)	2	9	28	0.833 (0.746, 0.908)	19	15	6	0.338 (0.239, 0.452)
11	37	3	0	0.038 (0.008, 0.106)	0	5	35	0.938 (0.881, 0.979)	12	20	8	0.450 (0.343, 0.565)
12	27	11	0	0.145 (0.076, 0.244)	1	10	27	0.842 (0.755, 0.916)	18	18	4	0.488 (0.380, 0.602)
13	32	6	0	0.079 (0.030, 0.164)	1	5	32	0.908 (0.838, 0.962)	14	17	9	0.438 (0.332, 0.553)
**Total**	**271**	**42**	**2**	**0.073** (**0.054**, **0.096**)	**6**	**72**	**237**	**0.866** (**0.839**, **0.892**)	**148**	**128**	**44**	**0.338** (**0.301**, **0.376**)

The incidence of the putative resistance allele for F1565C was lower compared with those of the other putative resistance alleles (Table 3). For Season 2 samples, contingency analyses indicated that the frequencies of C allele of F1565C were not significantly different between sites (χ^2^ = 12.127, df = 7, *p* = 0.092) and neither was the frequency of the G allele of V1023G (χ^2^ = 9.140, df = 7, *p* = 0.235) which is consistent with the respective results for Season 1. The P allele of S996P showed significantly different frequencies between sites in Season 2 (χ^2^ = 23.078, df = 7, *p* = 0.001), also consistent with the results of Season 1. Specifically, the inner city sites—11, 12 and 13—showed a tendency towards higher frequencies of this allele.

We also compared season 1 and season 2 for those sites where data were available for both seasons (Site 2, Site 3, Site 6, Site 7, Site 8). HRM results were arranged into a three-way-contingency table (5 sites × 2 alleles × 2 seasons). For the F1565C mutation, the final model excluded all the three-way and two-way interactions and included only season and allele as main effects (G = 18.104, df = 17, *p* = 0.382). This indicates that the mutation frequencies did not vary between seasons and sites.

For V1023G, log linear analysis indicated no three-way association between season and site and allele frequency, and two way interactions with allele frequency were also not significant. The log linear analysis for S996P indicated only a significant two-way interaction between sites*allele in a final model which was not significant (G = 8.290, df = 9, *p* = 0.505); removing the two-way interaction significantly changed the model (G = 21.558, df = 4, *p* < 0.001) reflecting significant differences between the sites. Overall the frequency of the resistance allele in mosquitoes at Site 3 and Site 7 was higher than at the other sites across the seasons.

### 3.4. Linking Mutations to Resistance

#### 3.4.1. WHO Insecticide Resistance Bioassays

Three *Ae. aegypti* samples (Site 6, Site 8 and *w*MelYog) were evaluated following standard WHO methods and diagnostic doses for deltamethrin and permethrin [42]. The mortality percentage for each insecticide and localities are shown in Table 4, Table 6 and Table 8. In all cases, *Ae. aegypti* from the three samples met the WHO criteria of mortality for resistance [42] (*i.e.*, <90% mortality) to both pyrethroids: deltamethrin and permethrin. Samples exposed to permethrin showed less mortality than samples exposed to deltamethrin. There were no consistent differences in mortality between sites 6, 8 and *w*MelYog with respect to pyrethroid resistance (data not shown).

#### 3.4.2. Single Mutation Analyses

**Figure 3:**
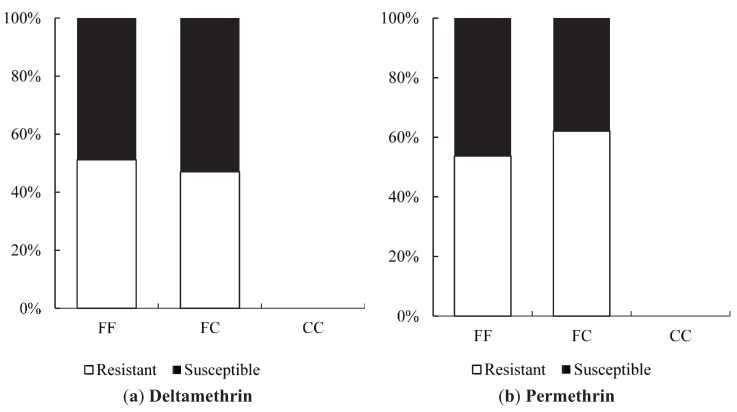
Association between *Vssc* F1565C mutation and resistance for *Aedes aegypti* from Yogyakarta (Site 6, Site 8 and *w*MelYog line). (**a**) Distribution of F1565C genotypes between deltamethrin-resistant and deltamethrin-susceptible mosquitoes. Only one individual of CC genotype was observed therefore this genotype is not presented (**b**) Distribution of F1565C genotypes between permethrin-resistant and permethrin-susceptible mosquitoes. Genotype CC was not observed in the permethrin assay.

We tested whether mutations influenced resistance to deltamethrin and permethrin by genotyping surviving and dead mosquitoes from WHO bioassays with HRM 1565, HRM1023 and HRM996. For the F1565C mutation, the frequency of the 1565C allele was low and when summed there was no clear difference in the frequency of this allele between the resistant and susceptible mosquitoes for either chemical (Figure 3). In total, the frequency of the putative resistance allele was 0.042 for deltamethrin resistant and 0.039 for susceptible individuals, while samples assayed with permethrin showed this allele at 0.075 in the resistant sample and 0.056 in the susceptible sample (Table 4). This was confirmed by the odds ratio which ranged from 0.66 to 4.11 with overlapping confidence intervals, though the ratio for permethrin in the *w*MelYog sample did show a marginally significant relationship between resistance and the heterozygote (Table 5). However, it should be noted that the F1565C mutation was hardly ever found in the homozygous form which might be expected to exhibit higher resistance than the heterozygote if the resistant allele is incompletely recessive.

**Figure 4:**
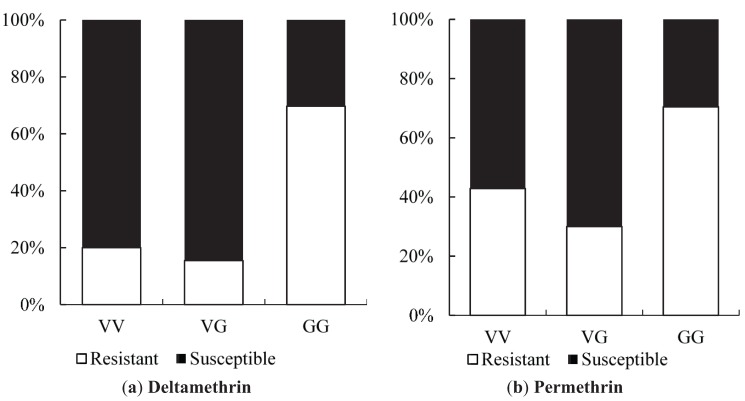
Association between *Vssc* V1023G mutation and pyrethroid resistance in *Aedes aegypti* from Yogyakarta (Site 6, Site 8 and *w*MelYog line). (**a**) Distribution of V1023G genotypes between deltamethrin-resistant and deltamethrin-susceptible mosquitoes. (**b**) Distribution of V1023G genotypes between permethrin-resistant and permethrin-susceptible mosquitoes.

**Figure 5:**
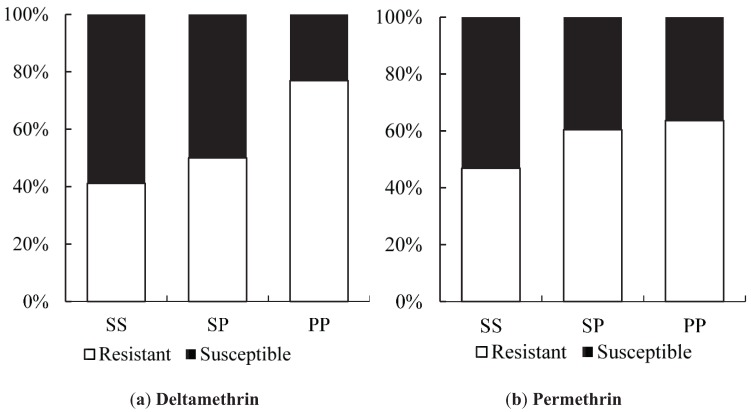
Association between *Vssc* S996P mutations and resistance to pyrethroids in *Ae*. *aegypti* from Yogyakarta (Site 6, Site 8 and *w*MelYog line). (**a**) Distribution of S996P genotypes between deltamethrin-resistant and deltamethrin-susceptible mosquitoes; (**b**) Distribution of S996P genotypes between permethrin-resistant and permethrin-susceptible mosquitoes.

In contrast, the V1023G mutation was found frequently in the homozygous state and was associated with resistance to pyrethroid insecticides. In all three samples for both chemicals, the frequency of the G allele was more abundant in the resistant individuals than the susceptible individuals and there are non-overlapping confidence intervals between samples in several instances (Table 6). Overall, 89.1% of the deltamethrin resistant mosquitoes from the WHO bioassay were mosquitoes that were homozygous for the mutant allele (G/G). 70% of individuals with genotype G/G were resistant to deltamethrin (Figure 4a). For the permethrin resistant samples, 71.0% of mosquitoes were homozygous for the mutant allele (G/G) and 70.5% of individuals with the genotype G/G were resistant (Figure 5b).

Resistance differences are also evident from the odds ratios (Table 7). When comparing the frequency of the two susceptible genotypes (VV and VG) with the resistant genotypes (GG) for deltamethrin resistance, the GG mutant genotype was more resistant than the VV or VG genotypes with an odds ratio overall of 9.50 (*p* = 0.002 from Fisher’s exact test), 10.50 (*p* < 0.001), and 22.00 (*p* < 0.001) for samples from Site 6, Site 8 and *w*MelYog respectively (Table 7). For permethrin resistance, the overall odds ratio was again similar for Sites 6 and 8 (6.07 *and* 5.68, respectively) and higher for *w*MelYog (8.57) and all were significant (Table 7).

**Table 4:** Genotypes and allele frequencies of *Vssc* mutation F1565C in *Aedes aegypti* mosquito samples from Yogyakarta (Site 6, Site 8 and *w*MelYog) assayed with deltamethrin and permethrin.

Population	% Mortality	Status	N	Total Insects Analysed by HRM	Genotypes			Freq. of C Allele (95% CI)
					FF	FC	CC	
**Deltamethrin**								
Site 6	35	R	131	40	38	2	0	0.025 (0.003, 0.087)
		S	70	33	29	4	0	0.061 (0.017, 0.148)
Site 8	71	R	63	40	37	3	0	0.038 (0.008, 0.106)
		S	152	40	38	2	0	0.025 (0.003, 0.087)
*w*MelYog	55	R	89	39	35	3	1	0.064 (0.021, 0.143)
		S	109	41	38	3	0	0.037 (0.008, 0.103)
Total	54	R	283	119	110	8	1	**0.042 (0.020, 0.076)**
		S	331	114	105	9	0	**0.039 (0.018, 0.074)**
**Permethrin**								
Site 6	21	R	158	40	36	4	0	0.050 (0.014, 0.123)
		S	42	28	25	3	0	0.054 (0.011, 0.149)
Site 8	16	R	169	40	36	4	0	0.050 (0.014, 0.123)
		S	31	31	26	5	0	0.081 (0.027, 0.178)
*w*MelYog	22	R	156	40	30	10	0	0.125 (0.062, 0.218)
		S	44	40	37	3	0	0.038 (0.008, 0.106)
**Total**	20	R	483	120	102	18	0	**0.075 (0.045, 0.116)**
		S	117	99	88	11	0	**0.056 (0.028, 0.097)**

**Table 5:** Association between homozygote and heterozygote *Vssc* F1565C alleles with *Aedes aegypti* mosquito susceptibility to permethrin and deltamethrin from Yogyakarta (CC genotype was rare and odds ratio could not be computed).

Population	Status	Total Insects Analysed by HRM	Genotype		Odds Ratio (95% CI)	*p* Value (Fisher’s Exact Test)
			FF	FC		
**Deltamethrin**						
Site 6	R	40	38	2	0.66 (0.112, 3.827)	NS *
	S	33	29	4		
Site 8	R	40	37	3	1.45 (0.243, 9.754)	NS *
	S	40	38	2		
*w*MelYog	R	39	38	1	1.09 (0.205, 5.738)	NS *
	S	41	41	0		
**Permethrin**						
Site 6	R	40	36	4	0.93 (0.190, 4.502)	NS *
	S	28	25	3		
Site 8	R	40	36	4	0.96 (0.198, 4.674)	NS *
	S	31	26	5		
*w*MelYog	R	40	30	10	4.11 (1.037, 16.295)	0.066
	S	40	37	3		

* NS: *p* > 0.10.

**Table 6:** Genotypes and allele frequencies of *Vssc* mutation V1023G in *Aedes aegypti* mosquito samples of Yogyakarta assayed with deltamethrin and permethrin.

Population	% Mortality	Status	N	Total Insects Analysed by HRM	Genotypes			Freq. of G Allele (95% CI)
					VV	VG	GG	
**Deltamethrin**								
Site 6	35	R	131	40	0	2	38	0.975 (0.940, 0.997)
		S	70	33	0	11	22	0.833 (0.738, 0.914)
Site 8	71	R	63	40	1	4	35	0.925 (0.863, 0.972)
		S	152	40	1	23	16	0.688 (0.584, 0.787)
*w*MelYog	55	R	89	39	1	5	33	0.910 (0.842, 0.963)
		S	109	41	7	26	8	0.512 (0.405, 0.624)
Total	54	R	283	119	2	11	106	**0.937 (0.904, 0.964)**
		S	331	114	8	60	46	**0.667 (0.605, 0.728)**
**Permethrin**								
Site 6	21	R	158	40	0	5	35	0.938 (0.881, 0.979)
		S	42	28	0	13	15	0.768 (0.653, 0.870)
Site 8	16	R	169	40	0	4	36	0.950 (0.899, 0.986)
		S	31	31	3	9	19	0.758 (0.647, 0.858)
*w*MelYog	22	R	156	40	3	15	22	0.738 (0.638, 0.830)
		S	44	40	1	34	5	0.550 (0.441, 0.662)
**Total**	20	R	483	120	3	24	93	**0.875 (0.831, 0.914)**
		S	117	99	4	56	39	**0.677 (0.610, 0.741)**

**Table 7:** Association between resistant and susceptible V1023G alleles with *Aedes aegypti* mosquito susceptibility to permethrin and deltamethrin.

Populations	Status	Total Insects Analysed by HRM	Genotype		Odds Ratio	*p* Value (Fisher’s Exact Test)
			VV or VG	GG	(95% CI)	
**Deltamethrin**						
Site 6	R	40	2	38	9.50 (1.927, 46.843)	0.002
	S	33	11	22		
Site 8	R	40	5	35	10.50 (3.390, 32.523)	<0.001
	S	40	24	16		
*w*MelYog	R	39	6	33	22.00 (6.863, 70.520)	<0.001
	S	41	33	8		
**Permethrin**						
Site 6	R	40	5	35	6.07 (1.835, 20.055)	0.004
	S	28	13	15		
Site 8	R	40	4	36	5.68 (1.611, 20.053)	0.009
	S	31	12	19		
*w*MelYog	R	40	18	22	8.56 (2.777, 26.358)	<0.001
	S	40	35	5		

The proportion of dead and surviving mosquitoes after exposure to insecticides for each genotype is shown in Figure 5.

In both chemicals assays, the individuals with the P/P mutant genotypes represented roughly 30% of the resistant individuals (Figure 5).

Results of HRM996 genotyping for the S996P mutation are shown in Table 8. Of the three mutations observed, the frequency of the putative resistance allele 996P ranged from 0.220 to 0.538 for deltamethrin and 0.213 to 0.463 for permethrin (Table 8).

**Table 8:** Genotypes and allele frequencies of *Vssc* mutation S996P in *Aedes aegypti* mosquito samples from Yogyakarta assayed with deltamethrin and permethrin.

Population	% Mortality	Status	N	Total Insects Analysed by HRM	Genotypes			Freq. of G Allele (95% CI)
					SS	SP	PP	
**Deltamethrin**								
Site 6	35	R	131	40	16	18	6	0.375 (0.273, 0.490)
		S	70	33	15	16	2	0.303 (0.199, 0.429)
Site 8	71	R	63	40	11	15	14	0.538 (0.429, 0.650)
		S	152	40	14	20	6	0.400 (0.296, 0.516)
*w*MelYog	55	R	89	39	10	19	10	0.500 (0.391, 0.615)
		S	109	41	24	16	1	0.220 (0.137, 0.325)
Total	54	R	283	119	37	52	30	**0.471 (0.408, 0.536)**
		S	331	114	53	52	9	**0.307 (0.249, 0.371)**
**Permethrin**								
Site 6	21	R	158	40	22	17	1	0.238 (0.152, 0.346)
		S	42	28	15	11	2	0.268 (0.162, 0.403)
Site 8	16	R	169	40	15	17	8	0.413 (0.308, 0.528)
		S	31	31	12	14	5	0.387 (0.271, 0.519)
*w*MelYog	22	R	156	40	8	27	5	0.463 (0.356, 0.578)
		S	44	40	24	15	1	0.213 (0.131, 0.318)
**Total**	20	R	483	120	45	61	14	**0.371 (0.311, 0.435)**
		S	117	99	51	40	8	**0.283 (0.222, 0.351)**

To determine whether this mutation confers resistance, the estimated odds ratio with 95% confidence and Fisher’s exact test for dead and surviving mosquitoes in each population were assessed (Table 9).

**Table 9:** Association between resistant and susceptible *Vssc* S996P alleles with mosquito susceptibility to permethrin and deltamethrin in Yogyakarta.

Populations	Status	Genotype		Odds Ratio	*p* Value (Fisher’s Exact Test)
		SS or SP	PP	(95% CI)	
**Deltamethrin**					
Site 6	R	34	6	2.735 (0.514, 14.569)	NS*
	S	31	2		
Site 8	R	26	14	3.051 (1.032, 9.022)	0.069
	S	34	6		
*w*MelYog	R	29	10	13.793 (1.671, 113.823)	0.003
	S	40	1		
**Permethrin**					
Site 6	R	39	1	0.333 (0.029, 3.867)	NS*
	S	26	2		
Site 8	R	32	8	1.300 (0.379, 4.454)	NS*
	S	26	5		
*w*MelYog	R	35	5	5.571 (0.620, 50.071)	NS*
	S	39	1		

* NS: *p* > 0.10.

There was no significant association between the S996P mutation and insecticide resistance for deltamethrin resistant individuals of Site 6 (odds ratio of 2.735 and *p* = 0.280) and Site 8 (odds ratio of 3.051 and *p* = 0.069), however odds ratios were greater than one and 95% confidence intervals were wide suggesting that a significant association may have been detected if sample sizes had been larger. Resistant individuals of *w*MelYog with the C/C mutant genotype showed a significant association with deltamethrin resistance (odds ratio of 13.793, *p* = 0.003). In all cases, there was no evidence from permethrin bioassay samples that individuals with the putative resistant genotype had any significant effect on permethrin resistance, with the odds ratio ranging from 0.333 to 5.571.

#### 3.4.3. Screening of *Wolbachia* Line *w*MelYog

F1565C, V1023G and S996P mutations were all found to be present in the laboratory *Wolbachia* line, *w*MelYog, at frequencies equivalent to those found at Sites 6 and 8 (Table 4, Table 5, Table 6, Table 7, Table 8 and Table 9). A putative resistant homozygote was observed in one individual of the *w*MelYog sample, and this individual was scored as resistant. There was an association between the F1565C heterozygote and permethrin survivors in this sample. In contrast, as already noted, mutation V1023G was commonly found in the homozygous state and was clearly associated with the resistant phenotype, while for mutation S996P there was also a significant association between the resistant genotype and the putative resistance allele for deltamethrin, but not permethrin in the *w*MelYog sample.

#### 3.4.4. Mutations in Combination

**Figure A2:**
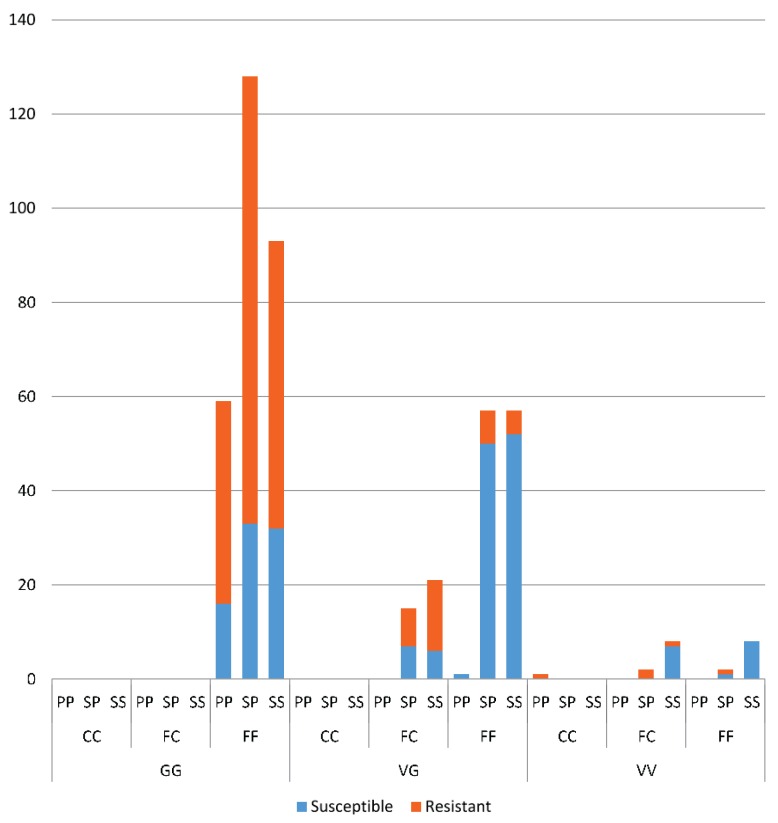
Genotypes of *Vssc* mutations V1023G, F1565C and S996P in order in individual *Aedes aegypti* from bioassay samples, Yogyakarta, Indonesia.

**Figure A3:**
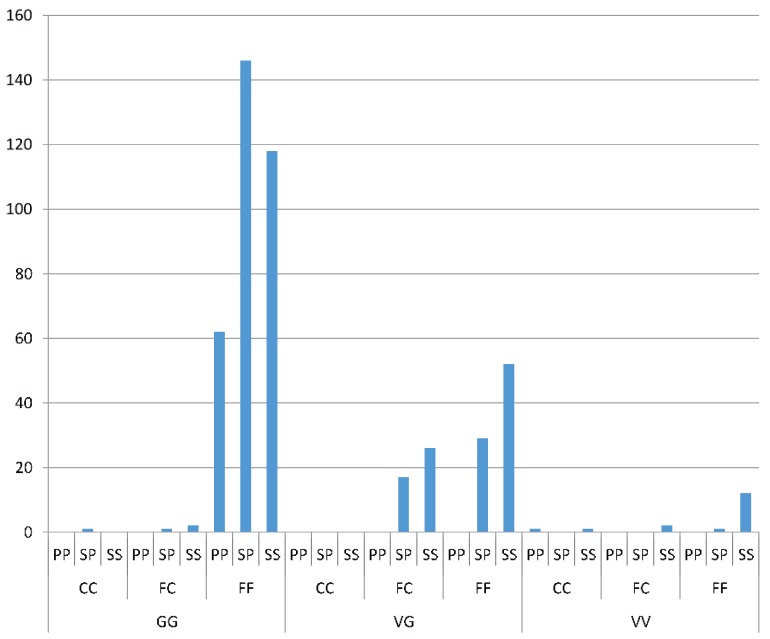
Genotypes of *Vssc* mutations V1023G, F1565C and S996P in order in individual *Aedes aegypti* from field samples, Yogyakarta, Indonesia.

Thirteen of a possible 27 genotypes with mutation combinations were observed in the mosquito bioassay samples (Table 10, Figure A2) and seven of these were common. Field samples of *Ae*. *aegypti* revealed 15 genotypes, four of which were not found in the bioassay samples, but the same seven mutation combinations were common (Figure A3). There was no individual homozygous for all three mutations found in either sample.

Individuals with heterozygous genotypes for all three mutations were found at a low frequency, but in equal numbers in the dead and survivor pools. F1565C/V1023G and F1565C/V1023G/S996P combinations were not found in the resistant homozygous state in any individuals in this study (GGGG and GGGGCC). The V1023G mutation was found alone in a large number of individuals (TTGGTT) and was more common in the survivors than in the dead mosquitoes for both chemicals. S996P was not found alone in the homozygous state (TTTTCC) and only two individuals were found alone in the heterozygous state, one scored as resistant and the other as susceptible to deltamethrin. One mosquito showed F1565C/S996P (GGTTCC) in the homozygous state and survived exposure to deltamethrin. The most common co-occurrence of the mutations was V1023G homozygous/S996P heterozygous (TTGGTC). The mutations V1023G/S996P co-occurred as homozygotes in some mosquitoes (TTGGCC). Odds ratios were used to determine whether mutations in combination were any more likely to be resistant than the V1023G homozygote alone, using the data from the permethrin and deltamethrin bioassays (Table 11). No comparison was made between homozygotes and heterozygotes because the degree of dominance for the heterozygote is unclear.

**Table 10:** Co-occurrence of *Vssc* mutations F1565C (T/G), V1023G (T/G) and S996P (T/C) in *Aedes aegypti* from Yogyakarta, Indonesia, Site 6, Site 8 and *w*MelYog line.

**Permethrin**														
Genotype	TT	TT	TT	TG	TG	TT	TT	TT	GG	TG	TG	TT	TT	Total
	GG	GG	GG	TG	TG	TG	TG	TG	TT	TT	TT	TT	TT	
	CC	TC	TT	TC	TT	CC	TC	TT	CC	TC	TT	TC	TT	
Alive	14	48	31	5	10	0	6	3	0	2	1	0	0	120
Dead	7	16	12	4	2	1	20	29	0	0	5	0	3	99
Total	21	64	43	9	12	1	26	32	0	2	6	0	3	219
**Deltamethrin**														
Genotype	TT	TT	TT	TG	TG	TT	TT	TT	GG	TG	TG	TT	TT	Total
	GG	GG	GG	TG	TG	TG	TG	TG	TT	TT	TT	TT	TT	
	CC	TC	TT	TC	TT	CC	TC	TT	CC	TC	TT	TC	TT	
Alive	29	47	30	3	5	0	1	2	1	0	0	1	0	119
Dead	9	17	20	3	4	0	30	23	0	0	2	1	5	114
Total	38	64	50	6	9	0	31	25	1	0	2	2	5	233
**Permethrin + deltamethrin**														
Genotype	TT	TT	TT	TG	TG	TT	TT	TT	GG	TG	TG	TT	TT	Total
	GG	GG	GG	TG	TG	TG	TG	TG	TT	TT	TT	TT	TT	
	CC	TC	TT	TC	TT	CC	TC	TT	CC	TC	TT	TC	TT	
Alive	43	95	61	8	15	0	7	5	1	2	1	1	0	239
Dead	16	33	32	7	6	1	50	52	0	0	7	1	8	213
Total	59	128	93	15	21	1	57	57	1	2	8	2	8	452

Fourteen other possible genotype combinations were not observed in the bioassay samples.

**Table 11:** Chance of *Aedes aegypti* being resistant to a pyrethroid insecticide ((**a**). permethrin Type I and (**b**) deltamethrin Type II) if multiple *Vssc* mutations are present (data from Yogyakarta Site 6, Site 8 and *w*MelYog line).

**(a) Permethrin**			
**Mutation Combination (F1565C, V1023G, S996P)**	**% Mortality**	**Odds Ratio (95% C.I.)**	***p***
TTGGCC/TTGGTT	33.3/27.9	0.77 (0.251–2.386)	NS
**(b) Deltamethrin**			
**Mutation Combination (F1565C, V1023G, S996P)**	**% Mortality**	**Odds Ratio (95% C.I.)**	***p***
TTGGCC/TTGGTT	23.7/40.0	2.15 (0.841–5.487)	NS

NS: *p* > 0.10.

No large resistance advantage to permethrin was conferred to the V1023G homozygote by addition of S996P in the homozygous form under the conditions of this study and with the sample sizes available. However, the odds ratio for homozygous mutations for 1023G and 996P in combination compared with 1023G and S996 with deltamethrin were greater than one suggesting some positive effect of the mutation for this Type II pyrethroid. None of the odds ratios was significant and all spanned 1, so the reliability of this conclusion is low, but mortality data also support this general trend (Table 11).

## 4. Discussion

Knockdown resistance in *Ae. aegypti* against pyrethroid insecticides may be conferred by one or more mutations present in the target site, the *Vssc* locus [18]. Genotyping of mutations directly related to insecticide resistance could provide a useful surveillance tool for monitoring resistance and helping to target chemical applications for vector control. Tetra-primer PCR assays for *Ae. aegypti* larvae were used to confirm a mutation at position 1565 in IIIS6 region of the *Vssc* in samples from Yogyakarta, Indonesia. The resistance allele frequency of 1565C observed (2.9% in Yogyakarta Season 1 and 10.9% in Yogyakarta Season 2) was lower than the frequency recorded elsewhere in South East Asian countries including Vietnam (21.6%) [26], Thailand (20%–100%) [38] and Myanmar (21.2%) [46]. The homozygous genotype, 1565C, was rare in mosquitoes sampled from Yogyakarta.

Sequence analysis of IIS6 region of *Vssc* from 75 larvae of *Ae. aegypti* collected in Yogyakarta revealed the presence of two mutations, V1023G and S996P. We detected a high frequency of the 1023G homozygotes (83%), and a moderate frequency of 996P homozygotes (17%). The V1023G mutation has been reported in other southeast Asian countries including Thailand [27,38,47] (allele frequency of 23%), Vietnam [26] (at a very low frequency), Myanmar [46] (80% homozygous) and Singapore [48] (44% homozygous). The simultaneous occurrence of both *kdr* mutations, V1023G and S996P, appears to be widely distributed in this region. The V1023G mutation has been found previously in Indonesia in the Semarang strain as first reported by Brengues *et al.* [18].

The large differences detected between the susceptible 1023V homozygotes and the heterozygote VG/homozygote GG sequences may be evidence of a genetic sweep of the 1023G resistance allele and proximate intron sequences in Yogyakarta. The sweep may be relatively recent because high rates of recombination have been observed in codon 1023 [23], but sequence variation within each of the three phenotypes is low. Similar evidence for a genetic sweep was detected in *Ae. aegypti* in Latin America and involved the alternative mutation 1023I [23].

Based on sequencing results, we have successfully developed RT-PCR HRM assays to genotype the V1023G, S996P and F1565C mutations. The assays initially were used to screen samples of *Ae*. *aegypti* from Yogyakarta Season 1 and Yogyakarta Season 2. In both seasons a high frequency of V1023G, a medium frequency of S996P and a very low frequency of F1565C were detected. This trend in frequencies is similar to that found in Myanmar [46], but not in Vietnam [26] or Singapore where the F1565C mutation can occur at high frequency [48], or in Thailand where there is fixation in some regions [38].

Odds ratio calculations to link mutations to insecticide resistance in three Yogyakarta populations indicated that the 1023G homozygote was more highly associated with both deltamethrin and permethrin resistance than susceptible heterozygotes, showing odds ratio values ranging from 5.68 to 22.00. Our result is in agreement with the findings of Du *et al.* [24], who were able to demonstrate that V1023G is one of the six mutations located in IIS6 that reduced the channel sensitivity to both deltamethrin (Type II) and permethrin (Type I) when expressed in *Xenopus* oocytes.

While 1023G is clearly associated with resistance, it does not completely explain the resistance phenotype in the Yogyakarta samples as there were 1023V homozygotes and heterozygotes which survived in the assay, as well as 1023G homozygotes which did not survive. Alternative mechanisms of resistance and/or differences in genetic background may be involved, and/or the WHO doses may also not be infallibly diagnostic of resistance. A previous study has shown that *Ae. aegypti* of the Indonesian Bandung strain were resistant to permethrin and deltamethrin with a RR_90_ of 79.3 and 23.7 respectively and suggested that detoxifying enzymes were involved as indicated by a high level of enzyme activity (oxidases, esterase A, esterase B) measured in a biochemical assay [16].

We found a suggestion of a synergistic effect of the S996P mutation when it occurred in conjunction with V1023G, particularly in relation to the Type II pyrethroid, deltamethrin which is similar to the findings of Hirata *et al.* [37]. We did not find the S996P mutation without V1023G as a homozygote in any individuals in the bioassay, but one individual was found in the field. When looking at S996P in isolation, in terms of frequency of the putative resistant allele, there was also suggestive evidence of an association with resistance to deltamethrin. Within the strain *w*MelYog and the mosquitoes from Site 8, there were high odds of a mosquito being resistant if it had the S996P mutation. Why Site 6 did not show a similar trend is unclear, but it would be worth investigating effects of different concentrations of insecticide to improve the diagnostic dose. The changes in S996P allele frequency between sites in Season 2 may point to ongoing selection.

Overall, the study demonstrates the presence of putative resistance alleles in *Ae*. *aegypti* in Yogyakarta. It also shows that at least one allele is associated with pyrethroid resistance and is, therefore, useful in ongoing monitoring of mosquito populations destined for release. The study also highlights the lack of strong differences in allele frequencies between sites around Yogyakarta, although there was a tendency for the inner city sites to have a higher frequency of resistance alleles for V1023G and S996P. These results suggest that in future releases of *Wolbachia*-infected mosquitoes, source populations with a suitable resistance background might be obtained from various sites around the city in a similar proposal as that made by Hoffmann *et al.* [40] for collection and dispersal of *Wolbachia*-infected mosquitoes from field-established “nurseries”.

## 5. Conclusions

A real time PCR-based HRM assay method was developed to detect F1565C, V1023G and S996P mutations in the voltage-sensitive sodium channel gene of *Ae*. *aegypti*. Each mutation was present in *Ae*. *aegypti* in sites around Yogyakarta, Indonesia, with V1023G occurring at the highest frequency. An association between resistance to permethrin and deltamethrin was identified for the V1023G mutation. The F1565C mutation was generally not associated with resistance, but this is likely to be because it was extremely rare in the homozygous form which is expected to be the most resistant genotype [28]. S996P conferred no apparent pyrethroid resistance advantage to the V1023G homozygote when present in either the homozygous or heterozygous form, but did show some association with deltamethrin resistance.

Bioassay results demonstrated that pyrethroid insecticides are likely to be losing efficacy in Yogyakarta and resistance management tactics should be employed. The low frequency of the F1565C mutations in the samples may indicate that Type I pyrethroids have not been used as extensively as Type II. Studies of metabolic resistance should also be conducted in Yogyakarta to understand fully the nature of resistance in *Ae*. *aegypti* in this city. A *Wolbachia*-infected laboratory strain with a Yogyakarta field genetic background (*w*MelYog) was shown to be equivalent in resistance status by bioassay and resistance allele frequency to the mosquitoes from the field at the sites where the original backcross mosquitoes had been collected. This strain should therefore be useful for general release around the city as part of a dengue suppression program.
